# Development of in-airway laser absorption spectroscopy for respiratory based measurements of cardiac output

**DOI:** 10.1038/s41598-021-84649-0

**Published:** 2021-03-04

**Authors:** Nicholas M. J. Smith, John Couper, Graham Richmond, Dominic Sandhu, Gus Hancock, Peter A. Robbins, Grant A. D. Ritchie

**Affiliations:** 1 Department of Chemistry, Physical and Theoretical Chemistry Laboratory, University of Oxford, Oxford, OX1 3QZ UK; 2 Department of Physiology, Anatomy and Genetics, University of Oxford, Oxford, UK

**Keywords:** Cardiology, Optics and photonics

## Abstract

Respiratory approaches to determining cardiac output in humans are securely rooted in mass balance and therefore potentially highly accurate. To address existing limitations in the gas analysis, we developed an in-airway analyser based on laser absorption spectroscopy to provide analyses every 10 ms. The technique for estimating cardiac output requires both a relatively soluble and insoluble tracer gas, and we employed acetylene and methane for these, respectively. A multipass cell was used to provide sufficient measurement sensitivity to enable analysis directly within the main gas stream, thus avoiding errors introduced by sidestream gas analysis. To assess performance, measurements of cardiac output were made during both rest and exercise on five successive days in each of six volunteers. The measurements were extremely repeatable (coefficient of variation ~ 7%). This new measurement technology provides a stable foundation against which the algorithm to calculate cardiac output can be further developed.

## Introduction

The accurate measurement of cardiac output, Q̇, in humans has proven to be a far less tractable problem than it may at first appear^1^. The ‘reference’ technique is generally considered to be that based on an application of mass balance originally proposed by Fick^2^. This technique requires both pulmonary and arterial catheters, relying on the relationship between Q̇, the arterio-venous blood oxygen content difference, and oxygen consumption measured from respired gas. As the accurate measurement of oxygen consumption in the critical care setting has been difficult, a much more widely used approach in that scenario has been that of indicator dilution. This avoids the measurement of oxygen consumption, but still requires pulmonary arterial catheterisation. In the early twenty-first century, safety concerns related to pulmonary arterial catheterisation arose, and subsequent studies found that their use was not associated with any improvement in mortality^3^. The use of pulmonary arterial catheters subsequently declined dramatically, and the measurement of Q̇ has moved towards less invasive approaches.

One non-invasive approach is to measure the rate of uptake of a tracer amount of acetylene from the breath. This approach was introduced by Grollman in 1928^4^ and became the ‘reference’ technique prior to the development of right-heart catheterisation^5,6^. Like the direct Fick and indicator dilution methods, Grollman’s technique is based on mass balance. For many years a closed-circuit implementation was used, where the uptake of acetylene could be determined from the change in its concentration in a bag from which the subject rebreathed. However, closed-circuit rebreathing methods are more awkward to use than their open-circuit counterparts, and furthermore they affect alveolar/arterial Pco_2_ and Po_2_ levels. Such considerations led to the development of open-circuit methods^7,8^ using mass spectrometers for the continuous acetylene analysis. The capacity of such sidestream analysers to measure breath-to-breath gas exchange is typically limited by difficulties associated with obtaining accurate measures of total flow, and time-aligning these with gas concentration measurements made following transportation from the principal path of gas flow.

The aim of the present study was to improve the measurement technology associated with the open-circuit acetylene approach for measuring Q̇. In 2016, Ciaffoni et al. developed new technology for measuring the respiratory exchange of CO_2_ and O_2_ that yielded a more than tenfold improvement in precision over existing techniques^9^. The approach used laser absorption spectroscopy to provide a highly precise, nearly instantaneous measure of gas composition in the respired gas flow, and the knowledge of this composition in turn allowed a highly precise determination of gas flow. The instrument capable of making these contemporaneous measurements was termed the molecular flow sensor (MFS). Here, we extend that approach by developing laser absorption spectroscopy to measure the uptake of trace amounts of acetylene and methane, and introducing this into a MFS device. As a proof of principle, a human study is conducted to determine the precision of cardiac output measurements obtained with the device.

## Results

### Development of acetylene and methane spectrometers

Acetylene has a well-resolved absorption spectrum in the near-infrared which lends itself to quantification by absorption spectroscopy using a diode laser. For this application, we chose a laser operating at 1.53 µm to probe a rovibrational transition. Methane was chosen as a relatively insoluble reference tracer gas, another species that absorbs well within the same region. A laser operating at 1.65 µm was used to probe a methane absorption feature that results from three overlapping transitions. These two spectral regions are sufficiently close that both laser sources could be multiplexed through the same optical fibre.

**Figure 1:**
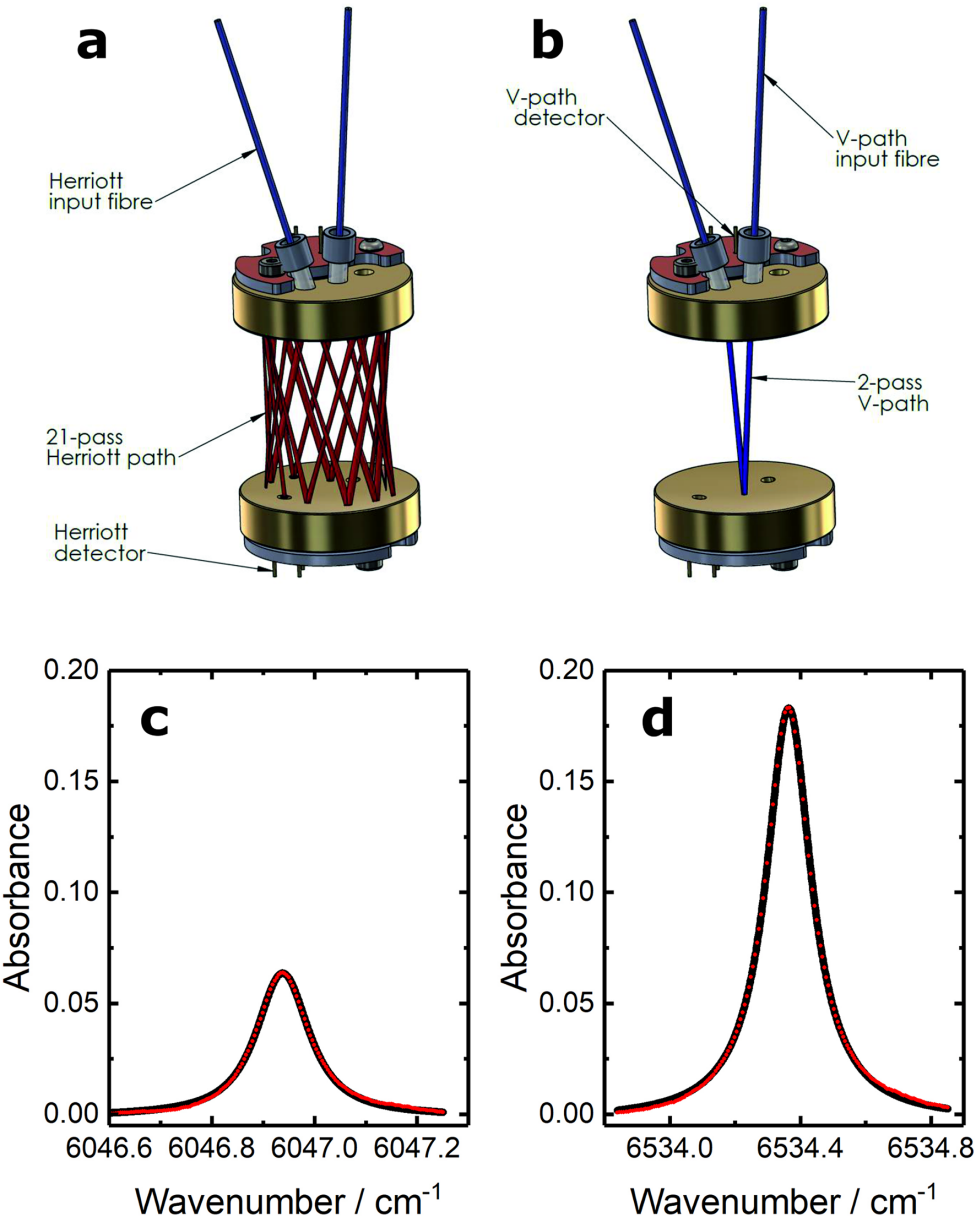
Implementation of multipass Herriott cell within the molecular flow sensor. (**a**) Schematic of the Herriott cell system. The spatially multiplexed radiation for probing CH_4_ and C_2_H_2_ is injected into the cell via a collimator, it follows a 21-pass optical path, and it is then detected by the photodiode labelled ‘Herriott detector.’ (**b**) Schematic illustrating the incorporation within the Herriott cell of the simple optical v-path used to probe CO_2_ and H_2_O. (**c**,**d**) Absorbance spectra from the Herriott cell are shown for 3030 ppm methane (**c**) and 2760 ppm acetylene (**d**) in synthetic air. Measured absorption spectra were obtained over 10 ms and are shown as filled red circles. The fitted Voigt distributions are shown as black lines.

As a target, we chose to employ tracer concentrations of 3000 ppm for acetylene and 9000 ppm for methane—the threefold difference arises because of a threefold difference in absorption cross-section between the two molecules. To obtain the required sensitivity, a spherical Herriott cell was designed and constructed, illustrated in Fig. 1a. The optical path length is extended with the radiation reflected a number of times, travelling back and forth through the sample gas^10^. The cell featured 20 reflections, yielding an optical path length of 56.7 cm. Furthermore, this design allows the incorporation of a V-path^9^ to measure CO_2_ and water concentrations (shown in Fig. 1b) without sacrificing sensitivity. The measurement of O_2_ at the same spatial position is also unaffected. Further details of the cell design are provided in “Methods”.

Following the successful implementation of the Herriott cell, it was integrated into the 3-gas (CO_2_, O_2_ and water vapour) MFS that provides highly accurate measurements of respiratory flow, total gas pressure, and gas temperature^9^. The result was a 5-gas MFS, also capable of providing precision measurements of acetylene and methane. Absorption spectra for each species were recorded at a frequency of 200 Hz and averaged to provide a spectrum every 10 ms. Example spectra recorded whilst a nominal 3000 ppm methane, 3000 ppm acetylene in synthetic air mixture flowed through the MFS device are shown in Fig. 1c,d, respectively. The excellent signal-to-noise ratio reflects the significant absorption that results from the extended optical path. The Voigt distribution fits these spectra extremely well, with number densities of the two species derived using the Beer-Lambert law. These number densities are then used to calculate the fractional concentration of each species. The minimum detectable concentrations were determined to be 48 ppm for methane and 15 ppm for acetylene, obtainable at 100 Hz. An overall precision of 1.6 and 0.6% is achieved for measurements of 3000 ppm mixtures of methane and acetylene, respectively.

**Figure 2:**
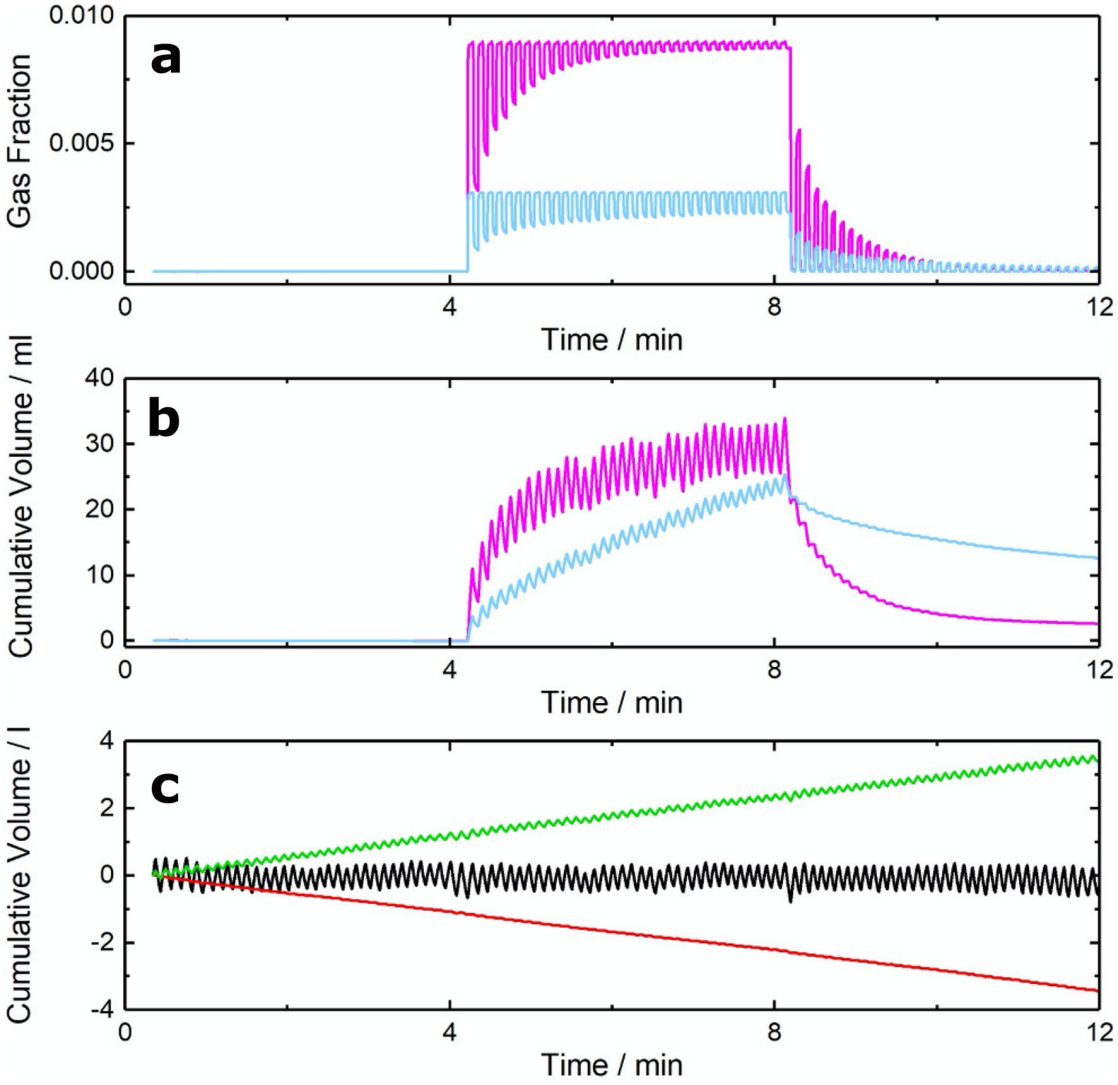
Gas fractions and cumulative gas exchange recorded before, during and after a tracer gas wash-in. (**a**) Gas fractions for methane (magenta) and acetylene (blue). (**b**) Cumulative uptake of methane (magenta) and acetylene (blue). (**c**) Cumulative gas exchange for oxygen (green), nitrogen (black), and carbon dioxide (red).

To evaluate the performance of the new 5-gas MFS, an experiment was undertaken with a volunteer breathing through the device with their nose occluded. Following 4 min of air-breathing, the inspiratory gas was changed to a mixture nominally containing 9000 ppm methane and 3000 ppm acetylene in a balance of synthetic air. This mixture was breathed as the inspiratory gas for a period of 4 min before returning to air again for the final 4 min. The results are shown in Fig. 2. During the tracer gas wash-in phase, the expiratory gas fraction for methane approaches that of the inspired gas mixture more rapidly than acetylene (Fig. 2a). This reflects the larger solubility of acetylene in the blood. During the washout phase, a similar behaviour is observed with the expiratory gas fraction of methane dropping quickly, whilst acetylene continues to be removed from the dissolved stores in the body. The solubility differences are also apparent within the tracer gas cumulative volume profiles (Fig. 2b). These profiles are the result of direct integration of the product of the measured flow and species concentration over time. The rate of acetylene uptake remains consistent throughout the wash-in period whilst the methane in the inspiratory mixture equilibrates more rapidly with the gas in the lung, slowing the rate of uptake. Throughout the procedure, the O_2_ consumption and CO_2_ production remained consistent (Fig. 2c). The same is true for the nitrogen, where the absence of any significant uptake or output illustrates the success of the flow measurement system.

### Human dataset

Data were collected for a group of six healthy male volunteers (21–40 years) in order both to develop the algorithm for deriving Q̇ and to provide an initial data set for comparison with literature. In order to assess within-person variability, each volunteer visited the laboratory on 5 consecutive days, with measurements made at rest and during light (50 W) exercise on each visit.

**Figure 3:**
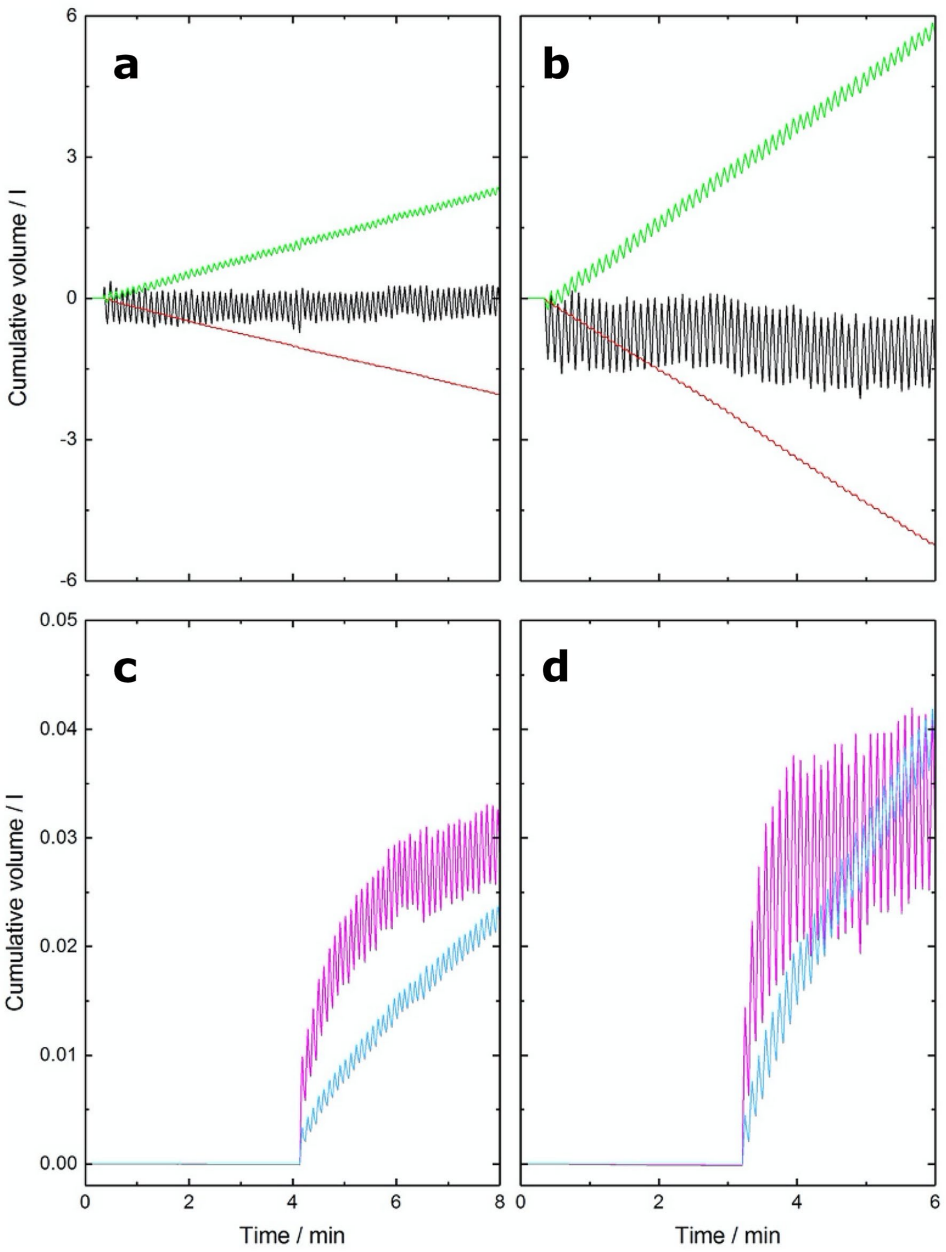
Example datasets for measurement of cardiac output at rest (left) and during exercise (right). (**a**,**b**) Cumulative gas exchange at rest (**a**) and during exercise (**b**) for oxygen (green), carbon dioxide (red), and nitrogen (black). (**c**,**d**) Cumulative uptake at rest (**c**) and during exercise (**d**) for methane (magenta) and acetylene (blue). The data illustrate the greater oxygen consumption, carbon dioxide production and tidal volumes during exercise. Total methane uptake is comparable at rest and during exercise, whilst acetylene uptake is significantly greater during exercise.

Figure 3 shows an example record of the tidal gas flow for each gas for one volunteer for rest and exercise. Points to note are: (1) The relative stability of the N_2_ record where there is no net-uptake or output from the body; (2) The larger tidal swings associated with the larger breaths that occur during exercise as compared with rest; (3) The approximately threefold greater O_2_ uptake and CO_2_ output during exercise compared with rest, as indicated by the steeper slopes of the O_2_ and CO_2_ records; (4) The difference in wash-in profile for methane versus acetylene, where the uptake of acetylene continues while that of methane slows because of their differing solubilities in blood.

### Development of algorithm to estimate Q̇

The accurate estimation of Q̇ from respiratory data required development of an algorithm which utilised data from the initial phase of the tracer gas wash-in. Within this algorithm, tracer gas uptake occurs by three mechanisms: equilibration with gas in the lung, uptake into lung tissue, and uptake into blood. The volumes of methane and acetylene taken up by the body at an adjusted functional residual capacity (FRC) are modelled and compared with recorded values. The estimated Q̇ and lung volume are those which minimise the sum of the squared differences between the modelled and measured tracer gas volumes. The assumptions inherent to this algorithm are now outlined, with further detail provided in “Methods”.

**Figure 4:**
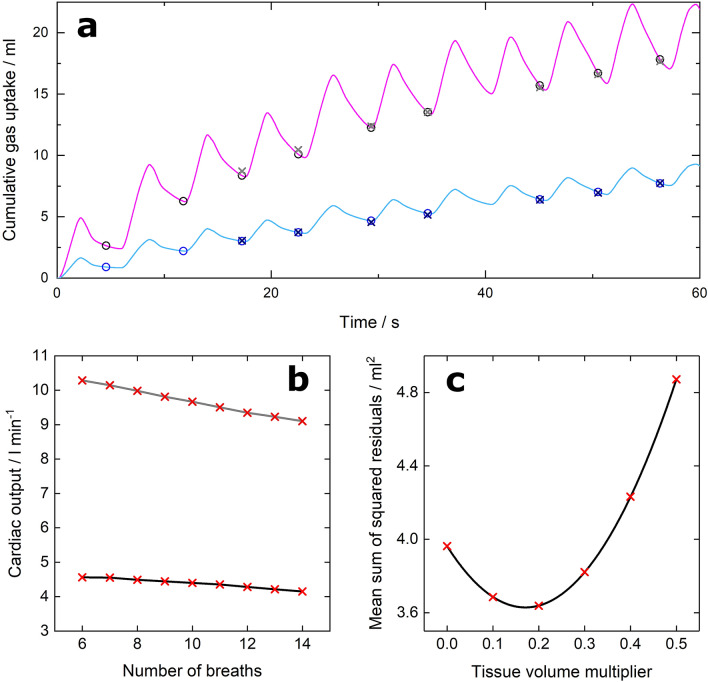
Calculation of cardiac output. (**a**) Example record of first 60 s of gas uptake for methane (magenta) and acetylene (blue). Symbols (methane, black; acetylene, blue) indicate values at the ends of modified breaths chosen so that lung volume is the same at each time point. Circles are data, and crosses are values predicted from the model. Note that there are no symbols associated with the breath ending close to 40 s as expiration was incomplete. The modified breath then becomes a double breath. (**b**) Average overall cardiac output (rest, black line; exercise, grey line) as a function of the number of breaths included in the analysis. (**c**) Mean sum of squared residuals across the 57 collected datasets as a function of lung tissue volume, expressed as a fraction of functional residual capacity. The optimal fraction was ~ 0.2 where the mean sum of squared residuals was at a minimum, showing good agreement with the value taken from the literature^11^.

The time points when the lung is at a constant standardised volume, the adjusted FRC, are indicated on representative acetylene and methane uptake records by the circles in Fig. 4a. If there is incomplete emptying of the lung, then no point is identified and the modified breath may be comprised of more than one actual breath. This is the case for the modified breath spanning either side of the 40 s time point in Fig. 4a.

The measured end-tidal partial pressures for acetylene and methane are used to infer both the concentrations of the gases in the lung, and the profiles for the partial pressures with which the blood is equilibrating as it flows through the lung. This enables a regression for the uptakes of acetylene and methane to be formed with the end-tidal values as the input, and with the lung volume and Q̇ as parameters to be estimated. The predicted uptakes from the regression are shown in Fig. 4a with crosses, generally lying very close to the measured data.

A limited number of breaths from the initial phase of the wash-in is used to estimate Q̇ because, after that, the amount of acetylene returning to the lung via the blood will become significant. Precisely how many breaths to include in the regression is arbitrary. We excluded the first two breaths because inhomogeneity within the lung means that the associated end-tidal measurements are unlikely to reflect overall lung concentration accurately. To explore the total number further, we plotted the relation between the number of breaths included in the estimation process and the average values obtained for Q̇, which is shown in Fig. 4b. For both rest and exercise the calculated Q̇ decreases with increasing breath number. In the further analysis, we limit the calculations to the first 10 breaths of the wash-in period.

A final consideration is that the gas remaining in the lung may either be in the gas phase or dissolved in the lung tissue. We used a standard value of 0.2 for the volume for lung tissue as a fraction of the end-expiratory lung volume^11^. To explore this assumption, we calculated the mean sum of squared residuals across the totality of the datasets with this fraction set to different values in the range 0 to 0.5. The results are shown in Fig. 4c and demonstrate that the best overall model fit is consistent with the value of 0.2 derived from the literature.

### Validation of cardiac output values

Of 60 experimental procedures performed, only three failed to return usable data. In two of these cases, irregular breathing patterns during the initial wash-in period led to the inability to identify a reasonable adjusted FRC. A third dataset was excluded due to significant rebreathing during the wash-in period of an exercise procedure. Thus, 57 datasets remained for analysis.

**Figure 5:**
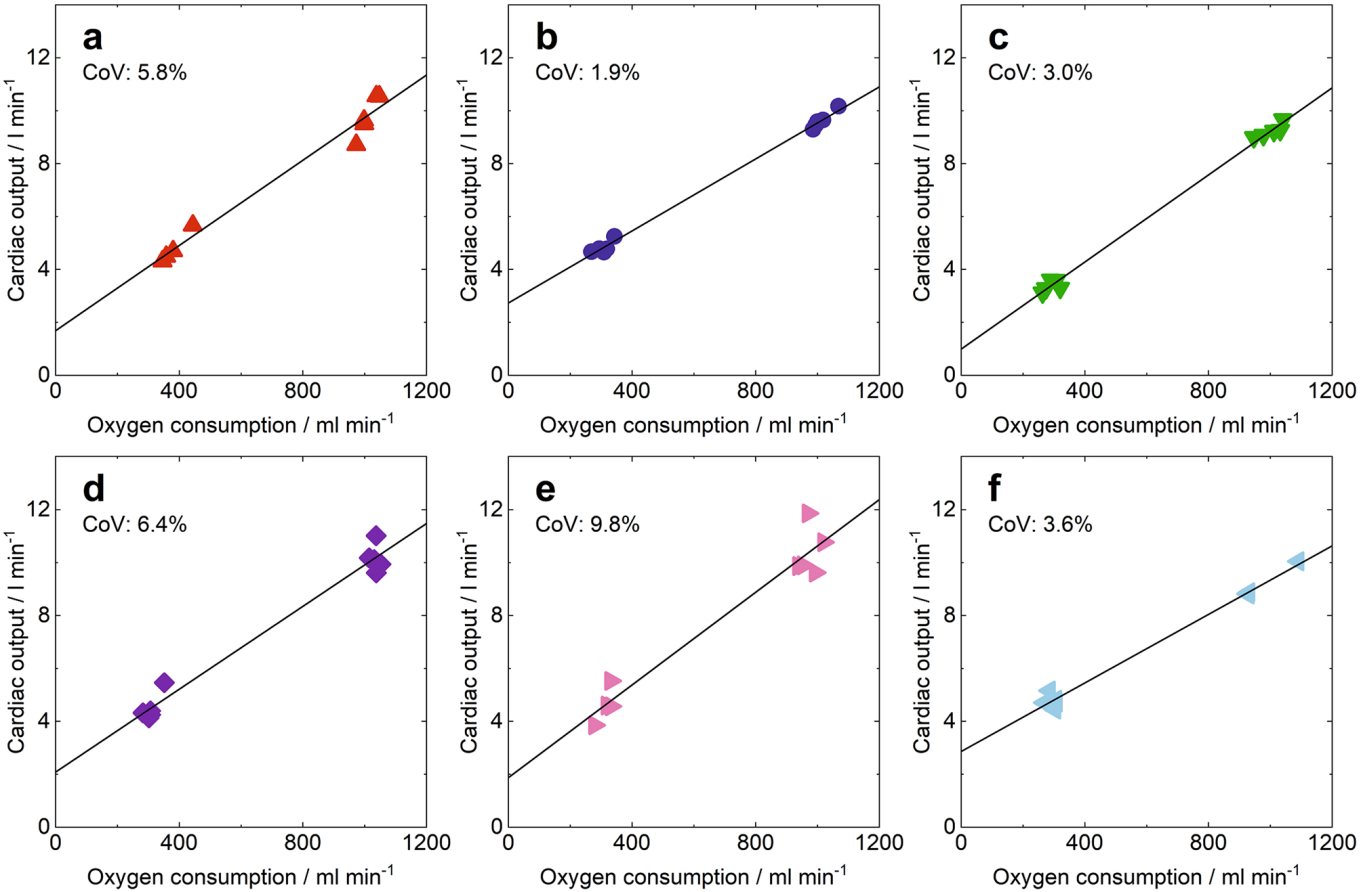
Intraparticipant relationships between cardiac output and oxygen consumption. (**a**–**f**), Cardiac output measurements are plotted against measured oxygen consumption for each participant in a separate panel, with intraparticipant linear fits shown with solid black lines. The quality of the fits is reflected in the coefficients of variation (CoV); below 10% of the mean cardiac output for every participant.

**Figure 6:**
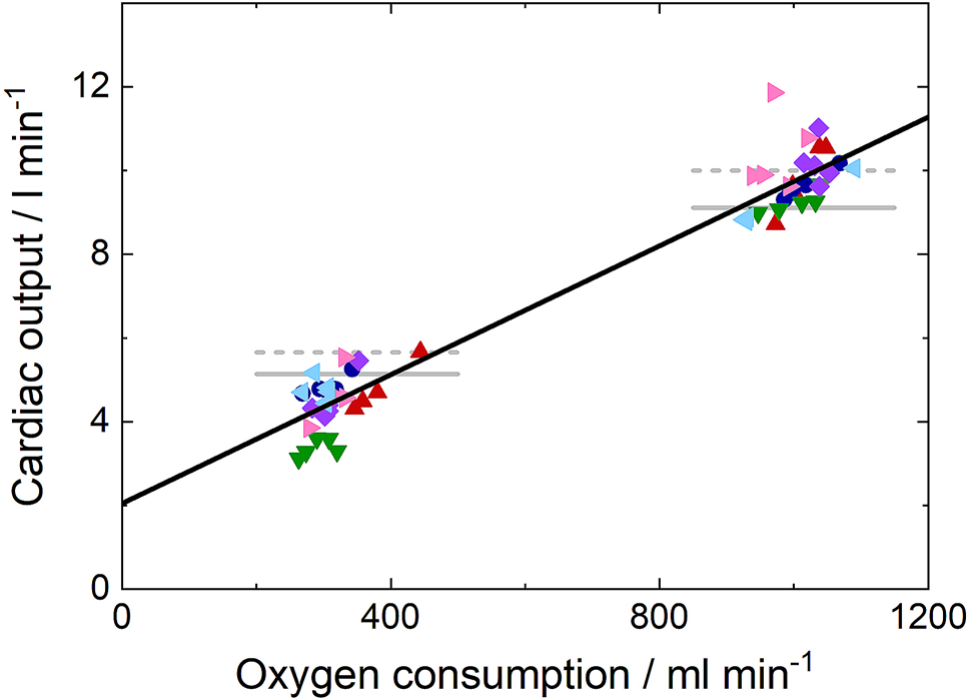
Fitted linear relationship between cardiac output and oxygen consumption. The linear fit to the measured cardiac output data is shown with a solid black line. Individual cardiac output measurements are shown with a different symbol and colour for each participant. Horizontal lines illustrate the predicted cardiac output values for the average measured oxygen consumptions at rest and during exercise from two studies using the direct Fick method. Data from Johnson *et al*.^12^ are shown as full lines, and those from Bevegard *et al*.^13^ as broken lines.

Values recovered for Q̇ from each dataset for each subject during rest and exercise are plotted against oxygen consumption, V̇o_2_, in Fig. 5. Mean values for V̇o_2_ and Q̇ for each participant are given in Table 1. Analysis of variance (ANOVA) was performed on the individual values for Q̇ with participant as a random factor, V̇o_2_ as a co-variate, both without and with the inclusion of the interactive term (participant * V̇o_2_). The former gave the mean regression slope (see Fig. 6) and the latter gave regression slopes for the individuals (see Fig. 5 and Table 1). The residual mean squared error of 0.243 l min^−1^ gives an estimate for the test to test variability and is 7% of the mean value for Q̇.

**Table 1:** For each participant, the measured oxygen consumptions (V̇o_2_) and determined cardiac outputs (Q̇) at rest and exercise are given (mean ± SD).

Subject number	V̇O_2_ rest/ml min^−1^	Q̇ rest/l min^−1^	V̇O_2_ ex/ml min^−1^	Q̇ ex/l min^−1^	*m*/10^–3^	*c*/l min^−1^	CoV/%
1	382 ± 44	4.79 ± 0.60	1011 ± 31	9.79 ± 0.78	8.06 ± 0.46	1.68 ± 0.37	5.8
2	307 ± 28	4.83 ± 0.24	1013 ± 33	9.63 ± 0.33	6.81 ± 0.12	2.73 ± 0.09	1.9
3	291 ± 23	3.38 ± 0.21	1002 ± 39	9.24 ± 0.26	8.23 ± 0.17	0.99 ± 0.12	3.0
4	310 ± 25	4.51 ± 0.54	1035 ± 14	10.17 ± 0.52	7.83 ± 0.41	2.08 ± 0.31	6.4
5	315 ± 25	4.63 ± 0.69	975 ± 32	10.40 ± 0.92	8.76 ± 0.78	1.87 ± 0.59	9.8
6	294 ± 17	4.76 ± 0.26	969 ± 81	9.14 ± 0.61	6.48 ± 0.24	2.86 ± 0.16	3.6
Group	317 ± 33	4.47 ± 0.67	1001 ± 25	9.75 ± 0.72	7.69 ± 0.23	2.05 ± 0.17	8.5

The gradient (*m*) and offset (*c*) of linear fits to each participant’s data are shown (mean ± SE), with the coefficient of variation (CoV) stated in each case. The parameters shown for the group, including CoV, describe a linear regression to all 57 data points. The overall CoV (within subject, between day) for this technology was found to be 6.9%.

Values for Q̇ were also determined using a previously published algorithm (OpCirc1)^12^. With this algorithm, the mean for Q̇ at rest was 2.7% higher, whilst during exercise it was 6.1% lower.

While it was not possible to obtain direct measurements of Q̇ with a pulmonary arterial catheter in our volunteers, there are nevertheless a number of reports in the literature giving regression relations between Q̇ measured using the direct Fick approach and V̇o_2_^12,13^. Figure 6 illustrates the predicted cardiac outputs for rest and exercise from these studies, allowing comparison with the measured data and regression relation obtained in this work.

## Discussion

There is a plethora of indirect techniques for determining Q̇^14–17^. Each is associated with limitations, but another reason behind the range of different technologies is that requirements differ between applications. For example, monitoring of Q̇ during anaesthesia requires a technology capable of near continuous measurement of cardiac output, such as thoracic impedance or Doppler ultrasonography. More generally, important considerations include safety, convenience, validity, precision, and accuracy.

The present study has developed a technology that falls within the general class of those based on respiratory exchange^16^. These technologies have the significant advantage that the calculation of Q̇ is based upon mass balance of the respiratory gases. Thus, their potential to give precise and accurate determinations is considerable when compared with, for example, a technique based on variations in transthoracic impedance, where there will always be uncertainty around the calibration factors required to transform these variations into measures of Q̇.

The development of open-circuit approaches^7,8^ has added substantially to the convenience of a respiratory-based measurement, whilst also removing the potential that closed-circuit approaches have to perturb Q̇ itself. However, these approaches still required a mass spectrometer to measure the acetylene and a reference gas with the same sample-line delay and dynamics. These devices, simply because of their cost, complexity, maintenance and calibration requirements, have never been adopted outside of a few specialist centres. Furthermore, the general lack of overall accuracy associated with open-circuit techniques for determination of breath-to-breath gas exchange would have applied to tracer gas uptake in these studies as it has done to measurements of respiratory gas exchange more generally^18^.

Recently, Ciaffoni *et al.* introduced the use of laser absorption spectroscopy across the main respired gas flow for the major gas species that are naturally exchanged across the lung^9^. This not only provided a far more convenient instrument than a respiratory mass spectrometer, it also provided a greater than tenfold increase in precision for the integration of the flow of specific gases into and out of the lung over time. This increase in precision arose in part because of the physical basis of the measurement (absorption spectroscopy), but also because the measurements were made completely contemporaneously (no sampling catheter) with those of flow in the mainstream gas under precisely the same physicochemical conditions. The present study has extended the path length for spectroscopic measurement across the main gas stream enabling measurement of tracer gas concentrations. The sensitivity has enabled a reduction in acetylene concentration from either 0.7%^12^ or 0.5%^19^ to < 0.3%, a considerable advantage given that a number of individuals can taste acetylene at concentrations above 0.5%^20^. The sensor was incorporated into the instrument of Ciaffoni *et al.*^9^ and consequently, the flow of the tracer gases can be integrated with the same overall precision as for the main components of the gas.

Measurement of Q̇ in healthy humans using MFS technology resulted in an overall coefficient of variation, CoV, (within subject, between day) of 6.9%. This demonstrates that the device is capable of highly-precise measurements, especially given that some of the overall variation will be real physiological variation. A detailed assessment for the CoV for the thermodilution technique (which employs a pulmonary arterial catheter) suggests that a value of 25% is appropriate for a single measurement, and a standard procedure has been to repeat the measurement three times in order to gain a value of ~ 15%^21^. Values are available for other techniques^15,22^, and while generally lower than for thermodilution, they generally do not relate to a between-day assessment.

Ethical considerations relating to pulmonary arterial catheterisation prevented us from comparing our results with simultaneous measurements of Q̇ obtained using the direct Fick technique. Nevertheless, during exercise our results compared very favourably with those reported previously by Johnson *et al.*^12^ and Bevegard *et al.*^13^ (Fig. 6). At rest, however, our values mostly fell below the mean values predicted from these previous studies.

These differences between studies may reflect genuine physiological differences between the participants. Brandfonbrener *et al.* demonstrated using the dye dilution technique that the standard deviation for basal cardiac output measurements between subjects was very large, at 30% of the mean value across a group of 60 participants^23^. Furthermore, in the study by Bevegard *et al.*^13^, the participants were recruited from a pool of blood donors and had a mean haemoglobin concentration of 13.2 g dL^−1^, compared with a mean value of 15.4 g dL^−1^ for the present study. As reductions in haemoglobin concentration are associated with increases in cardiac output^24^, we should expect the values for Q̇ obtained by Bevegard *et al.* to be higher.

If the differences in resting Q̇ between our study and those using the direct Fick technique are not physiological, then the most likely explanation is that the algorithm for recovering Q̇ from the tracer gas measurements is biased at rest. Specifically, the algorithm assumes: (i) that the lung can be represented as a single well-mixed volume; (ii) that gas concentration in the overall lung volume can be reasonably approximated by interpolation between end-tidal values; and (iii) that recirculation of gas back into the lung can be discounted. All of these assumptions are flawed. Swanson^25^ demonstrated that, for breath-to-breath variations in pulmonary gas exchange, the effective lung volume was significantly smaller than the true lung volume. This arises from inhomogeneity of ventilation to volume ratios through the lung. Busso *et al*.^26^ demonstrated that changes in end-tidal composition generally overestimate the real changes in gas concentration within the lung. Finally, the initial rise in tracer gas fraction in the blood returning to the lung can be quite rapid as a result of the highly uneven distribution of perfusion between the different organs of the body. For example, the total renal mass for humans is ~ 300 gm, but the blood flow to the kidneys is ~ 20% of the entire cardiac output and this is associated with a transit time through the entire systemic circulation of ~ 10 s. Overall, inhomogeneities in lung function may cause estimates of Q̇ made from the first breath or two of uptake to be particularly inaccurate, while recirculation will progressively reduce the accuracy associated with measurements made during the later breaths. Indeed, this second point is well illustrated by the dependency of Q̇ on the number of breaths included in the analysis (Fig. 4b). Finally, these limitations are not confined to the algorithm employed in this study, but apply equally to the algorithms of others such as the OpCirc1 algorithm of Johnson *et al.*^12^.

Apart from their non-invasive nature, the reason behind continuing to develop respiratory techniques for estimating Q̇ relates to their grounding in mass balance, and therefore their potential to deliver values that are not only highly repeatable and precise, but also accurate and unbiased. To deliver such results, potential sources of bias within the existing algorithms need to be removed. One possible way forward is suggested by the work of Mountain *et al*.^27^, who developed an approach to identify parameters describing person-specific lung inhomogeneities from the wash-in or washout of an inert gas. Their approach also incorporated a rudimentary model of body gas stores for nitrogen^28^ to address recirculation. The findings from that study raise the possibility of simultaneously estimating Q̇ and lung inhomogeneity in a manner that allows for the presence of recirculation using the wash-in of acetylene and methane. Such an algorithm should be substantially more accurate than those currently available that assume a uniform lung and no recirculation. The technological developments of the present study now provide a sound platform on which to base such future work.

In conclusion, this study has succeeded in developing a far more precise and convenient measurement technology for a respiratory approach to determining cardiac output. The associated human study demonstrates that the technology enables a precision of measurement that is probably greater than that achieved by any other method. However, despite good agreement with literature values during mild exercise, it appears that the measurement is potentially biased under resting conditions. Future work needs to be directed at algorithm development, in particular the removal of certain simplifying assumptions that have the potential to introduce measurement bias. The determination of the absolute concentrations of the trace gases depends, for an assumed path length, upon the accuracy of the integrated absorption cross-sections as given in the HITRAN database. Reduced uncertainties in these measured quantities would aid in reducing uncertainties in such future measurements.

## Methods

### Development of methane and acetylene spectrometers

A pair of distributed feedback (DFB) diode lasers operating within the near-IR region were used as radiation sources to evaluate the concentrations of methane and acetylene by direct laser absorption spectroscopy. The spectral window used to probe each species was chosen to provide sufficient sensitivity and to avoid interference from transitions arising from other major components of breath, principally carbon dioxide and water.

To evaluate acetylene concentrations, a 10 mW DFB diode laser (NTT Electronics, NLK1C5EAAA) operating at 1.53 µm was used to probe the P(8) transition within a vibrational combination band (ν_1_ + ν_3_). A second 10 mW DFB diode laser (NTT Electronics, NLK1U5EAAA) operating at 1.65 µm was used to probe the methane absorption feature resulting from three overlapping R(3) transitions within the first overtone band of one of the C–H stretching vibrations (ν_3_). This feature has previously been used to quantify atmospheric methane concentrations in field conditions^29^. The pair of lasers was housed within an electronics module, along with the laser chip temperature and applied current control circuits.

To provide sufficient sensitivity for accurate evaluation of the tracer gas concentrations, a measurement cell based on a spherical Herriott cell was developed. In these cells, radiation is reflected a number of times, in a well-defined spatial pattern, by high reflectivity spherical concave mirrors. In this way, the optical path length is extended with the radiation travelling back and forth through the sample gas. Significant path lengths can be achieved in a low volume with little loss of optical power, leading to the implementation of multipass optical cells in a range of portable field systems and airborne applications, particularly in combination with sensitivity enhancing phase-sensitive detection^30^. As shown in Fig. 1, the light enters and exits the optical cell via strategically placed holes on the mirrors^10^. The nature of the spot pattern within a Herriott cell is dependent on a number of factors: the radius of curvature of the mirrors, the separation of the mirrors, and the angle and position of the radiation injection. When correctly aligned, the spot pattern, describing the positions of the reflections on the pair of mirrors, can be circular or elliptical. The optical and mechanical stability of Herriott cell-based systems has been found to be superior to other similar multipass optical systems, such as White cells^31^.

The chosen cell design was based on modelled reflection spot patterns calculated using a ray propagation model described by Tarsitano and Webster^31^. The model assumes that the beam propagates as a paraxial ray, a small reflection angle ray tracing approximation^32^. The design selected used a mirror separation of 27.61 mm, each mirror with radius of curvature of 80 mm. Radiation entered the cell through an angled injection aperture in the first mirror and is then reflected between the mirror surfaces, before exiting the optical cell via an off-centre exit aperture on the second mirror. The optical path comprises 20 reflections, resulting in an overall path length of 56.7 cm. Radiation is ultimately detected with a photodiode placed behind the exit aperture in the second mirror. The spot pattern is contained well within the mirror surface diameter of 24 mm and both injection and exit apertures are positioned away from the edges of the mirror surfaces, allowing sufficient space to incorporate the collimator and photodiode housing packages. The series of reflections leads to successive focusing and defocusing of an optical beam. Large beam widths at reflection points neighbouring the injection and exit apertures could lead to signal loss and/or detection of radiation that has not traversed the entirety of the intended optical path. The optical beam was also modelled as a Gaussian beam, the width of which can be calculated using the complex beam parameter^33^, to ensure that these effects did not occur.

A further advantage of the cell design, beyond the extended optical path length, is the potential to facilitate additional gas sensing channels within the pair of spherical mirrors. Crucially for the target application in this work, there is sufficient space at the centre of the injection mirror to allow the inclusion of an optical v-path similar to that in the pre-existing MFS, with both injection and exit apertures on the extended path's input mirror. The schematic shown in Fig. 1a illustrates how the Herriott cell integrates into the pre-existing MFS reported by Ciaffoni *et al.*^9^. This integrated design allows sensitive measurement of methane, acetylene, carbon dioxide, and water concentrations within an MFS without negatively affecting the sensitivity of the device or increasing the device’s volume.

Mirrors with the specified diameter, radius of curvature, and aperture positions and angles were manufactured (LBP Optics) and were comprised of nickel-plated aluminium blanks, with the mirror surfaces gold coated by electroplating. The mirrors were housed in a measurement cell, with an InGaAs photodiode (Hamamatsu, G12181-020K) installed beneath the output mirror surface.

The two near-IR lasers were spatially multiplexed, using a 2 × 2 fibre coupler with a 50:50 coupling ratio (Thorlabs, PN1550R5A2), allowing radiation from both to share a single optical path. Once multiplexed, the lasers could be sequentially scanned across the absorption features. As one laser is scanned over an appropriate spectral window, the other ‘stationary’ laser is held at a constant current (and hence wavelength) above threshold. An ‘offset amplitude spectrum’ must be determined for each laser and subtracted before any data analysis is performed. The spectral ranges across which the lasers were scanned were evaluated using an optical spectrum analyser (Melles Griot, with a free spectral range of 0.067 cm^−1^), which provided the relative calibration to a precision of 0.3% (calculated from the uncertainty in measuring the spectrum analyser peak positions). The absolute wavelength was determined using a laser wavelength meter (Exfo WA-1000), and this allowed clear identification of the spectral features by comparison with the HITRAN database^34^.

Spectra for each species are recorded at a frequency of 200 Hz, with 200 data points recorded per spectrum. Two spectra were averaged, providing a spectrum every 10 ms. For the real-time fitting of the data the first 30 data points of each spectrum are excluded to allow the ‘stationary’ laser sufficient time to establish a constant ‘offset amplitude’ signal level. A theoretically predicted Voigt distribution is regressed onto each measured absorbance spectrum in real-time. Using molecular transition line strengths taken from the HITRAN database^34^ and the optical path length (calibrated using gas mixtures with defined methane and acetylene compositions), the fitted distributions enable evaluation of species number densities. Coupling these values with measurements of total gas pressure and temperature allows fractional gas concentrations to be determined. Temperature and pressure precisions were 0.07 and 0.10% respectively and were determined by comparing the instrument outputs with those measured with standard thermocouples and a high precision mercury barometer. These uncertainties were propagated linearly, giving a 0.17% uncertainty in gas fraction.

The minimum detectable concentration of each system is calculated as the species concentration that corresponds to a level of absorbance equal to one standard deviation of the residuals between the measured spectrum and the fitted Voigt distribution. The precision of the fitting to the assumed Voigt function proved the major source of uncertainty in our measurements. The minimum detectable concentrations were propagated in quadrature with the uncertainties associated with temperature and pressure measurements and that from the wavenumber axis calibration to evaluate the overall precision of the spectrometers. Prior to use, the multiplexed system is calibrated; offset and baseline spectra recorded with pure nitrogen flowing through the measurement cell at 5 l min^−1^.

### Modelling body tracer gas uptake

The clinical potential of the highly accurate contemporaneously measured gas compositions and respiratory flows relies on the computational model used to interpret the recorded data. A model of methane and acetylene uptake in the body was written in order to determine cardiac output, Q̇. Tracer gas uptake is assumed to occur by three mechanisms: equilibration with the gas in the lung (the tracer gas volume remaining in the lung at the end of each breath is equal to the product of the lung volume and end-tidal tracer gas fraction), uptake into the lung tissue (with a volume equal to the product of the lung tissue volume, end-tidal tracer gas fraction, and solubility of the tracer gas), and uptake by the blood (the volume of which in each breath is given by the product of gas solubility, Q̇, alveolar pressure of the tracer gas, and the duration of the breath). Thus, the basis of the uptake modelling is the following equation:

1 $${V}_{E}^{g}\left(i\right)={V}_{L}\hspace{0.17em}{F}_{E}^{g}\left(i\right)+{V}_{tiss}\hspace{0.17em}{\beta }^{g}\hspace{0.17em}k {F}_{E}^{g}\left(i\right)+\dot{Q}\hspace{0.17em}{\beta }^{g}\hspace{0.17em}{\sum }_{i}{P}_{A}^{g}\left(i\right)\hspace{0.17em}\{{t}_{E}\left(i\right)-{t}_{E}\left(i-1\right)\} ,$$

where *V*_*E*_^*g*^(*i*) is the cumulative uptake volume of gas *g* at the end of expiration of breath *i*, ${\text{V}_\text{L}}$ is the lung's gas volume at functional residual capacity (FRC), *F*_*E*_^*g*^(*i*) is the end-tidal gas fraction of gas *g* for breath *i*, *V*_*tiss*_ is the lung's tissue volume, *β *^*g*^ is the solubility of gas *g*, *k* is a constant conversion factor from a dry gas fraction to a pressure in units of atm under body temperature and pressure saturated (BTPS) conditions, *P*_*A*_^*g*^(*i*) is the alveolar pressure of gas *g* for breath *i*, and *t*_*E*_(*i*) is the time of the end-tidal point of breath *i*.

In order to evaluate the model at a consistent lung volume, for both gas and tissue, the model would ideally be evaluated when the lungs are at FRC. However, although end-expiratory volume approximates FRC, there is significant breath-to-breath variation in this quantity, making it difficult to identify a consistent volume at which the lungs are at FRC. To estimate where FRC is on the tidal volume record, a cubic smoothing spline is fitted to the end expiratory points for the total gas flow into and out of the lung. The lung will be close to FRC when this spline intersects this tidal volume record. Due to breath-to-breath tidal volume variations, however, there will be a large number of breaths with end-expiratory lung volumes greater than FRC. In order to provide the model with cumulative volume data from the majority of recorded breaths, an adjusted FRC volume is used. To address this, the fitted smoothing spline was translated upwards so that it intersected the volume record towards the end of expiration for most breaths. It was found that this translation was small, specified ultimately as one standard deviation of the residuals of the spline fit. The adjusted FRC indices are then identified as the times at which the shifted spline crosses the tidal volume record during expiration (descending volume). For breaths where the end-expiratory volume lies above this shifted spline, the expiration is judged incomplete and the following modified breath consists of data from two true breaths. Associated end-inspiratory points are identified as the maxima of the tidal volume record.

The gas fractions are directly measured by the 5-gas MFS, and the uptake volumes calculated as the cumulative integral of the product of the measured flow and gas fractions. The alveolar pressure of gas *g* for breath *i*, *P*_*A*_^*g*^(*i*), cannot be measured directly and so an average pressure during the breath is estimated using:

2 $${P}_{A}^{g}\left(i\right) =k\left[ \left\{{t}_{ins}\left(i\right)\left(\frac{{F}_{E}^{g}\left(i-1\right)+ {F}_{E}^{g}\left(i\right)}{2}\right)+{t}_{ex}\left(i\right){F}_{E}^{g}\left(i\right)\right\}\frac{1}{{t}_{breath}\left(i\right)}\right] ,$$

where *t*_*ins*_ (*i*) is the inspiratory time period of breath *i*, *t*_*ex*_(*i*) is the expiratory time period of breath *i*, *t*_*breath*_(*i*) is the total time period of breath *i*.

The solubility constant, *β*^*g*^, used in this work is a Bunsen coefficient, with units of ‘volume of gas at STPD per volume of fluid measured at atmospheric pressure and body temperature’^35^. Literature values for methane and acetylene Bunsen coefficients in blood display significant variation. The greater number of studies seeking to determine the acetylene solubility in human blood reflects the long-standing use of this gas within physiological experiments. The Bunsen coefficients for methane, *β *^CH4^, and acetylene, *β *^C2H2^, in blood used in this work are taken from Wagner^36^.

A number of assumptions are present within this model of tracer gas uptake. The first to note is that Q̇ and ${\text{V}_\text{L}}$ are assumed to be constants over the range of breaths used for analysis. Associated with this initial assumption is the fact that the model assumes a homogeneous lung. The ${\text{V}_\text{L}}$ parameter determined from this modelling is, in reality, an estimation of the lung volume into which the inspired tracer gas mixture is well mixed. In a lung with inhomogeneous ventilation, peripheral lung units may require multiple breaths before good mixing with the inspired tracer gas mixture is achieved. Inhomogeneity inherent within the lungs provides deviation from two further modelling assumptions. The first is that the calculated parameter *P*_*A*_^*g*^(*i*) represents an effective mean alveolar pressure for the period of the *i*th breath, and the second is that measured end-tidal fraction *F*_*E*_^*g*^(*i*) represents an effective end expiratory fraction within the lung.

The tracer gas solubilities in lung tissue volume are assumed to be the same as those used for blood, justified for acetylene by the similarity of the measured solubilities of this gas in blood and lung tissue^37^. Methane is a significantly less soluble species, so deviations from this assumption are unlikely to introduce significant error.

The model also makes no consideration of the recirculation of tracer gases in the blood. Both tracer gases are non-physiological gases and as such the blood concentrations are assumed to be zero before inspiration of a tracer gas mixture. During the tracer gas breathing period acetylene, and to a lesser extent methane, will be taken up by the blood. Mixed venous blood containing tracer gas will later return to the lungs. The recirculation of tracer gas-laden blood reduces the uptake of further gas from the lungs into the blood, and thus from the perspective of this model which does not take this into account, an associated reduction in estimated Q̇ would be predicted.

The model solution method adopted was multiple linear regression. The number of variables within the multiple linear regression was limited to two. This was achieved by introducing the constant tissue volume multiplier, *X*, such that the lung tissue volume, *V*_*tiss*_, is equal to ${\text{V}_\text{L}}$ × *X.* Tracer gas uptake expressions for the 3rd to 10th breaths of the tracer gas wash-in were used within the analysis. The first two breaths are excluded due to the significant effects of ventilation inhomogeneity within the lung in these early breaths; lung inhomogeneity isn't considered within the model. Data from breaths beyond the 10th were excluded to avoid the estimation utilising data in which the recirculation of the tracer gases into the lungs would contribute significantly.

### Human dataset

A proof of principle study was performed in order to assess the success of this non-invasive Q̇ determination technique, combining the highly-precise 5-gas MFS with the physiological model of methane and acetylene uptake. A group of volunteers were studied on 5 days, at rest and during exercise.

Six men, aged 21–40 years, who had no history of cardiac or respiratory illnesses, volunteered for the study. The experimental work was carried out in accordance with the general principles of the declaration of Helsinki, with ethical approval obtained from the South Central Oxford A Research Ethics Committee (reference number 17/SC/0172) and all participants providing written informed consent prior to the study.

Each volunteer visited the laboratory on 5 consecutive days. On each visit, tracer gas wash-ins were performed at rest and then during submaximal exercise. Experimental data were collected by the 5-gas MFS. An open-circuit breathing system was used, with inspiratory gas supplied to the volunteers using a flow past system at flow rates of 30 and 60 l min^−1^ for the rest and exercise procedures respectively. The inspiratory gas changed from air to a 9000 ppm methane, 3000 ppm acetylene, and 21% oxygen with a balance of nitrogen mixture for the tracer gas wash-in period. All experimental procedures were carried out with the volunteers seated on a bicycle ergometer, and consisted of a period of air breathing, followed by a tracer gas wash-in. At rest, these two periods each lasted 4 min, whilst at exercise each period was shortened to 3 min. Submaximal exercise was performed with the bicycle set to 50 W, with the volunteers asked to maintain a revolution rate of 60 rpm. Volunteers pedalled for 7 min prior to the start of data collection to ensure an elevated and approximately constant cardiac output throughout the recorded procedure.

Each of the recorded datasets was analysed using the model of tracer gas uptake by the body. Within this model a constant tissue volume multiplier, *X*, is defined, such that the lung’s tissue volume is equal to *X* multiplied by the determined lung volume. For each input dataset, the determined cardiac output and lung volume will therefore vary with the chosen tissue volume multiplier, *X*. In order to optimise the value of *X* used in this work, the quality of the model fits to the measured data were evaluated for a range of *X* values. *X* was varied between 0 and 0.5 in steps of 0.1 and the model was fit to the data by multiple linear regression for each of the datasets. To evaluate the optimal *X* value, the sum of squared residuals between simulated and measured tracer gas uptakes for all of the datasets is analysed. The trend was fitted with a quadratic function, with the *X* value at the minimum of this fitted function taken as the optimal tissue volume multiplier as shown in Fig. 4c. Estimated Q̇ parameters were then found for each dataset using this optimised tissue volume multiplier.

ANOVA was performed on the values for Q̇ with participant as a random factor, V̇o_2_ as a co-variate, both without and with the inclusion of the interactive term (participant * V̇o_2_). The former provided a mean regression slope, with the latter giving regression slopes for each individual. It is from these regression slopes that a residual mean square error is calculated to give an estimate of the error associated with the Q̇ parameters.

## Data Availability

All data needed to evaluate the conclusions in the paper are present in the paper. Additional data related to this paper may be requested from the authors.
